# Serum concentrations of canine pancreatic lipase immunoreactivity and C‐reactive protein for monitoring disease progression in dogs with acute pancreatitis

**DOI:** 10.1111/jvim.16218

**Published:** 2021-07-11

**Authors:** Kirstin M. Keany, Geoffrey T. Fosgate, Sean M. Perry, Shannon T. Stroup, Joerg M. Steiner

**Affiliations:** ^1^ MedVet New Orleans, Louisiana and MedVet Mandeville, Louisiana USA; ^2^ Department of Production Animal Studies, Faculty of Veterinary Science University of Pretoria Onderstepoort South Africa; ^3^ Department of Veterinary Services Mississippi Aquarium Gulfport Mississippi USA; ^4^ Gastrointestinal Laboratory, Department of Small Animal Clinical Sciences College of Veterinary Medicine and Biomedical Sciences, Texas A&M University College Station Texas USA; ^5^ Present address: VCA Animal Referral and Emergency Center of Arizona Mesa AZ USA

**Keywords:** albumin, biomarker, canine acute pancreatitis clinical severity index, modified canine activity index, survival

## Abstract

**Background:**

Reliable biomarkers for monitoring disease progression and management in dogs with acute pancreatitis have not been described.

**Objective:**

To determine if serum concentrations of canine pancreatic lipase immunoreactivity (cPLI) and C‐reactive protein (CRP) can be used as biomarkers for disease progression in hospitalized dogs with acute pancreatitis.

**Animals:**

Thirteen hospitalized dogs with acute pancreatitis diagnosed based on clinical signs, serum cPLI concentrations, and imaging findings were enrolled.

**Methods:**

Serum cPLI and CRP concentrations were determined before and then daily during hospital management and 1 week after hospital discharge. Modified canine activity index (MCAI) and canine acute pancreatitis clinical severity index (CAPCSI) scores were calculated daily for each patient while hospitalized.

**Results:**

The MCAI scores (*P* = .03) but not CAPCSI scores (*P* = .31) were significantly different between dogs that survived to discharge (n = 11) and those that did not (n = 2). Serum cPLI concentration was positively correlated with MCAI (rho = 0.42; *P* = .01). Serum CRP concentration also was positively correlated with the MCAI (rho = 0.42, *P* = .01).

**Conclusions:**

Serum cPLI and possibly CRP could be used as objective biomarkers for clinical changes in hospitalized dogs with acute pancreatitis. Additional studies involving larger numbers of dogs would be warranted to evaluate the broader impact of these findings.

**Abbreviations**
95% CI: 95% confidence interval
CAPCSI: canine acute pancreatitis clinical severity index
CAPS: canine acute pancreatitis severity score
CIBDAI: canine inflammatory bowel disease activity index
cPLI: canine pancreatic lipase immunoreactivity
CRP: C‐reactive protein
IBD: inflammatory bowel disease
MCAI: modified canine activity index
sCAPS: simplified version of the canine acute pancreatitis severity score
SIRS: systemic inflammatory response syndrome

## 1 INTRODUCTION

Diagnosis of acute pancreatitis in dogs is based on a combination of clinical signs, abdominal imaging findings, and measurement of serum markers.^1^, ^2^ Measurement of canine serum pancreatic lipase immunoreactivity (cPLI) concentration by Spec cPL has been reported to have sensitivity of 42% to 90.9%^2^, ^3^ and specificity of 74.1% to 100%^4^ for diagnosing pancreatitis. Serum C‐reactive protein (CRP) is a positive acute phase reactant synthesized by the liver that increases in response to pro‐inflammatory cytokines.^5^, ^6^ Serum CRP concentrations increase in several inflammatory, immune‐mediated, infectious, and neoplastic diseases in dogs.^7^, ^8^, ^9^, ^10^, ^11^, ^12^, ^13^, ^14^, ^15^, ^16^, ^17^, ^18^, ^19^, ^20^, ^21^

Acute pancreatitis in dogs can present with variable severity. Between 27% and 58% of dogs with acute pancreatitis die from the disease or from secondary complications.^22^, ^23^, ^24^, ^25^ Some affected dogs are slow to recover or appear stagnant in their clinical condition while hospitalized. This variability makes it difficult to determine prognosis and represents a financial burden on clients who can be unsure whether or not to continue management.

Previous studies have attempted to identify independent predictors of death in dogs with acute pancreatitis. One study described 2 scoring systems that were shown to predict short‐term mortality (ie, death within 30 days) of dogs with acute pancreatitis: the canine acute pancreatitis severity score (CAPS) and the simplified version of CAPS (sCAPS).^25^ The CAPS score incorporates the presence of systemic inflammatory response syndrome (SIRS), coagulation disorders, increased serum creatinine concentration, and ionized hypocalcemia. The CAPS score has a sensitivity of 89% and specificity of 90% for prediction of short‐term (ie, <30 days) death in dogs with acute pancreatitis. The sCAPS includes respiratory rate instead of SIRS,^25^ and has a sensitivity of 96% and a specificity of 77% for predicting short‐term death in dogs with acute pancreatitis. The modified canine activity index (MCAI) is based on the canine inflammatory bowel disease activity index (CIBDAI), which is an objective measure for assessing the severity of inflammatory bowel disease (IBD) in dogs. The index since has been successfully used for a wide variety of studies of IBD in dogs.^12^, ^26^, ^27^, ^28^ Because acute pancreatitis shares many clinical signs with IBD, the CIBDAI also was considered a potential study tool to assess dogs with acute pancreatitis and thus the CIBDAI has been modified for use in dogs with pancreatitis in some previous studies (Joerg M. Steiner, personal communication, 2021). In addition to serum concentrations of CRP and cPLI, we used both of these scoring systems to standardize the clinical evaluation of dogs with acute pancreatitis.

Our objective was to evaluate whether serum CRP and cPLI concentrations are useful biomarkers for assessing clinical changes in hospitalized dogs with acute pancreatitis. We hypothesized that serum CRP and cPLI concentrations would decrease during hospitalization in association with clinical improvement as dogs recovered from acute pancreatitis, as assessed by the MCAI and the canine acute pancreatitis clinical severity index (CAPCSI).

## 2 MATERIALS AND METHODS

### 2.1 Case enrollment

The study was approved by MedVet's research department and each client signed an informed consent document before patient enrollment. A prospective cohort study was performed for dogs that presented to the MedVet Emergency or Internal Medicine Service that were diagnosed with acute pancreatitis. Inclusion criteria included dogs that presented with ≥2 clinical signs associated with acute pancreatitis (eg, vomiting, diarrhea, hyporexia, anorexia, abdominal pain), had abdominal ultrasound examination findings consistent with pancreatitis (eg, hyperechoic mesentery with hypoechoic pancreas), and had serum cPLI concentration >400 μL/L at the time of presentation (day 0). Dogs were excluded if they had ≥2 previous episodes of acute pancreatitis (either suspected or confirmed), were suspected to have chronic pancreatitis, had serum cPLI concentration ≤ 400 μL/L, or were moderately or severely anemic or became moderately or severely anemic during the study (hematocrit <25%). Anemic dogs were excluded because of concerns regarding the necessity of repeated blood sample collection for the purpose of the study. Dogs also were excluded if serum samples were missing from any corresponding hospitalization or re‐evaluation day, or if the blood sample was mishandled or thawed at any time.

Enrolled dogs were hospitalized and medically managed, including a combination of the following: antiemetics (eg, maropitant, metoclopramide), antacids (eg, pantoprazole), analgesics (eg, buprenorphine, fentanyl, lidocaine, ketamine), IV fluids (eg, Plasma‐Lyte‐A, Normasol‐R, or lactated Ringer's solution), antibiotics (eg, metronidazole, enrofloxacin, ampicillin, sulbactam), and a nasogastric tube with a continuous rate infusion of a low fat liquid diet. Other treatments for individual patients with concurrent diseases included dexamethasone, prednisone, insulin (eg, porcine insulin zinc suspension, neutral protamine Hagedorn, human recombinant insulin), ophthalmic medications, and vitamin B12.

Of the 13 dogs enrolled, only 3 (23%) did not have a documented concurrent disease. Concurrent known disease processes within the 30 days before presentation or identified upon presentation to the clinic included thrombocytopenia, diabetes mellitus, lipemia, urolithiasis (struvite), intervertebral disc disease, valvular endocardiosis, diabetic ketoacidosis, azotemia, apocrine gland adenocarcinoma, and biliary mucocele. One dog (1/13) had a previous history of acute pancreatitis (single previous episode).

### 2.2 Clinical data collection

Modified canine activity index (Table 1) and CAPCSI (Table 2) were calculated daily by an emergency clinician (before transferring the patient to the internal medicine service) or by an internal medicine resident or staff internist. Whenever possible, the same observer performed the clinical scoring each day of hospitalization and during follow‐up treatment for each individual patient.

**TABLE 1:** Modified canine activity index (MCAI): The MCAI score utilizes 7 variables of clinical relevance in dogs with acute pancreatitis (AP), including activity, appetite (voluntary food intake), vomiting, cranial abdominal pain, dehydration, feces consistency, and blood in the feces based on the following categories

**Activity** 0 = Normal (as usual) 1 = Slightly decreased (the animal stands less than usual) 2 = Moderately decreased (the animal is reluctant to stand up) 3 = Severely decreased (the animal cannot stand up)	**Appetite (voluntary food intake)** 0 = Normal (the animal ate more than ¾ of the food offered) 1 = Slightly decreased (the animal ate about ½ of the food offered) 2 = Moderately decreased (the animal ate about ¼ of the food offered) 3 = Severely decreased (the animal did not eat much of the food or at all)
**Vomiting** 0 = None 1 = 1‐2 times/d 2 = 3‐4 times/d 3 = ≥5 times/d	**Cranial abdominal pain** 0 = None (no signs of pain) 1 = Mild (abdominal wall resistance or other signs of pain are elicited upon palpation of the abdomen, the animal moves slowly or is less responsive) 2 = Moderate (the animal resists palpation, is reluctant to move when encouraged, does not lie on its side) 3 = Severe (persistent vocalization, howling, and/or insomnia)
**Dehydration** 0 = None (<5%, no signs of dehydration) 1 = Mild (5%, slight loss of skin elasticity) 2 = Moderate (6%‐8%, decreased skin turgor, slight delayed capillary refill time, dry mucous membranes, sunken eyes) 3 = Severe (≥10%, severely decreased skin turgor, delayed capillary refill time, deeply sunken eyes, severely dry mucous membranes)	**Feces consistency** 0 = Normal (well formed) 1 = Soft (slightly watery and poorly formed) 2 = Diarrhea (no form and runny) 3 = Watery (watery with no solids and pale)
**Blood in the feces** 0 = Absent 1 = Present	

**TABLE 2:** Canine acute pancreatitis clinical severity index (CAPCSI): The CAPCSI score utilizes 4 variables of clinical relevance in dogs with AP, including cardiac, respiratory, intestinal integrity, and vascular forces based on the following categories

**Cardiac** 0 = No abnormalities 1 = >60 ventricular premature complexes/24‐h period or heart rate > 180 beats/min 2 = Paroxysmal or sustained ventricular tachycardia	**Respiratory** 0 = No abnormalities 1 = Clinical evidence of dyspnea or tachypnea (>40 breaths/min) 2 = Clinical evidence of pneumonia or acute respiratory distress syndrome
**Intestinal integrity** 0 = No abnormalities 1 = Intestinal sounds not detected during >3 auscultations in 24‐h period 2 = Hematochezia, melena, or regurgitation 3 = No enteral food intake for >3 d 4 = No enteral food intake for >3 d and at least 2 of the following: hematochezia, melena, or regurgitation	**Blood pressure** 0 = No abnormalities 1 = Systolic arterial blood pressure <60 or >180 mm Hg *or* serum albumin concentration <1.8 g/dL 2 = Systolic arterial blood pressure <60 or >180 mm Hg *and* serum albumin concentration < 1.8 g/dL

A minimum of 1 mL of serum was collected from each patient every day of hospitalization, and on follow‐up examination approximately 1 week after discharge. Serum was collected within 30 minutes of CAPCSI and MCAI scoring. Blood was drawn into a red top (nonserum separator) tube, and centrifuged at 1200*g* within 15 minutes of collection. Serum was separated and stored at −20°C within 15 minutes after centrifugation. Each tube was labeled with patient name, identification, and date. Day 0 corresponded with the first day of hospitalization and enrollment in the study. Each sample collected after day 0 was labeled accordingly, with the re‐evaluation sample labeled “recheck,” 7 to 10 days postdischarge from the hospital. The initial sample (day 0) was shipped frozen and on ice to the Gastrointestinal laboratory (GI Lab) at Texas A&M using overnight shipping to determine if the patient met the inclusion criteria of a serum cPLI concentration >400 μg/L. The remaining samples were shipped overnight frozen and on ice after the last re‐evaluation sample had been obtained for the final patient.

### 2.3 Biomarker evaluation

Serum samples for cPLI and CRP were processed at the GI Lab at Texas A&M University. Serum samples were stored at 80°C until processing. Serum cPLI concentration was measured using a commercial ELISA (Spec cPL, Idexx Laboratories, Westbrook, Maine) and concentrations >400 μg/L were considered diagnostic for pancreatitis. Serum CRP concentrations were measured using an ELISA (Tri Delta Diagnostics, Boonton, New Jersey). Serum concentrations >10 mg/L were considered to be abnormal.

### 2.4 Statistical analysis

The normality assumption was assessed by calculating descriptive statistics, plotting histograms, and performing the Anderson‐Darling test using a commercial software package (MINITAB Statistical Software, Release 13.32, Minitab Inc, State College, Pennsylvania). Data were displayed using scatter plots in the ggplot2 package^29^ within R.^30^ Nonparametric Spearman's correlation coefficients were estimated between evaluated biomarkers and clinical scores overall by incorporating data points from all dogs from the day of diagnosis until discharge or death. Linear mixed‐effects models were used to estimate nonparametric correlations, while adjusting for individual dog effects. All data were rank‐transformed before statistical analysis and models were fitted with clinical scores as the dependent variables and biomarkers as independent variables. The data set was restricted to observations from the day of diagnosis until discharge or death and dog was included as a random effect with a variance components correlation structure. Correlation statistics were estimated as the product of the regression coefficient for the biomarker multiplied by the ratio formed by the SD of the ranks from the biomarker divided by the SD of the clinical score ranks. Significance of the correlation was reported as the significance of the regression coefficient. Clinical scores also were compared between died/euthanized dogs and surviving dogs using similar mixed‐effects models, including survivor status as a fixed effect. Correlations were estimated and regression models fitted using a commercial software package (IBM SPSS Statistics Version 25, International Business Machines Corp., Armonk, New York) with significance set as *P* < .05.

## 3 RESULTS

The study was conducted from February 2018 to March 2019. Eighteen dogs met the inclusion criteria for the duration of the study. Five dogs were removed from the study because they were lost to follow‐up, or for inadequate sample handling, leaving 13 dogs for data analysis. The mean ± SD age of the study population was 9.5 ± 3.1 years (range, 3.4‐12.8 years). All dogs were neutered, and included 8 males and 5 females. Breeds included the following: 2 West Highland white terriers, 2 Chihuahuas, 2 Yorkshire terriers, and 1 each of Shih Tzu, Maltese mix, Cockapoo, Pomeranian, Dachshund, miniature poodle, and chow mix. The baseline (day 0) cPLI concentrations ranged from 407 to 4704 μg/L (mean 1739 μg/L) for survivors and from 1632 to 20 704 μg/L (mean 11 168 μg/L) for nonsurvivors. Clinical signs associated with acute pancreatitis on initial presentation (day 0) included vomiting (10/13; 77%), diarrhea (8/13; 62%), hyporexia (6/13; 46%), anorexia (7/13; 54%), and abdominal pain (8/13; 62%). None of these clinical signs appeared more or less commonly in survivors vs nonsurvivors.

Eleven dogs (85%; 95% confidence interval [CI], 58%‐97%) survived to discharge. Two dogs (15%; 95% CI, 3%‐42%) died during the study; 1 was euthanized on day 3 of treatment and the other died of unknown cause but was suspected to have died because of pulmonary thromboembolism based on clinical signs. However, the owner of this dog did not consent to necropsy.

**FIGURE 1:**
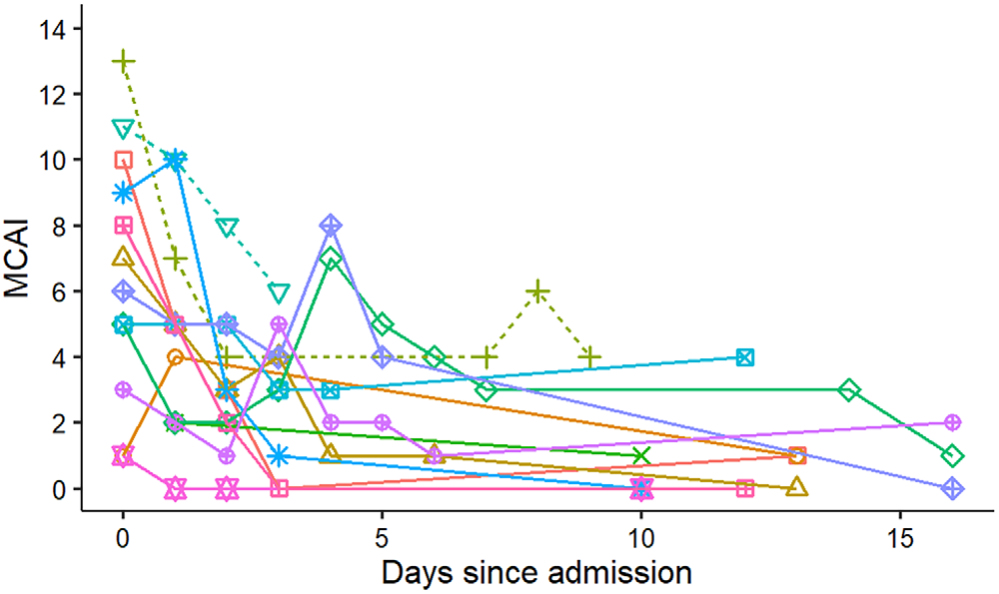
Change in modified canine activity index (MCAI) in 13 hospitalized dogs with pancreatitis from a single referral hospital. Solid lines symbolize surviving dogs and dashed lines indicate dogs that died or were euthanized

**FIGURE 2:**
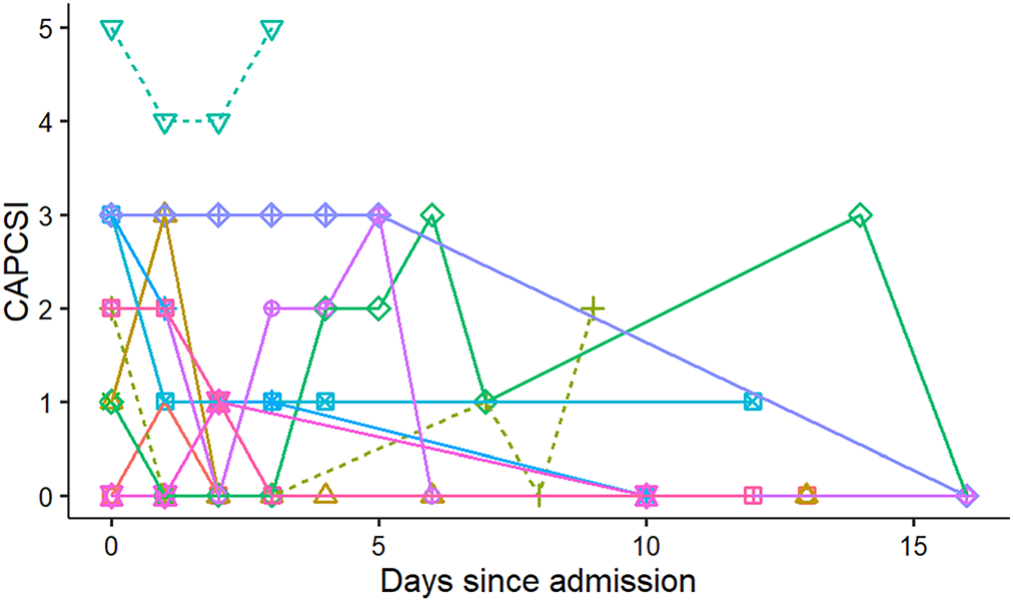
Change in canine acute pancreatitis clinical severity index (CAPCSI) in 13 hospitalized dogs with pancreatitis from a single referral hospital. Solid lines symbolize surviving dogs and dashed lines indicate dogs that died or were euthanized

The median (range) MCAI score during hospitalization (ie, not including the MCAI on re‐evaluation 7‐10 days after discharge) was 6 (4‐13) and 3 (0‐10) for died/euthanized and discharged dogs, respectively (*P* = .03). The median (range) CAPCSI during hospitalization was 2 (0‐5) and 1 (0‐3) for died/euthanized and discharged dogs, respectively (*P* = .31). The MCAI clinical scores decreased over time in enrolled patients (Figure 1), but a clear temporal pattern for CAPCSI scores was not apparent (Figure 2).

**FIGURE 3:**
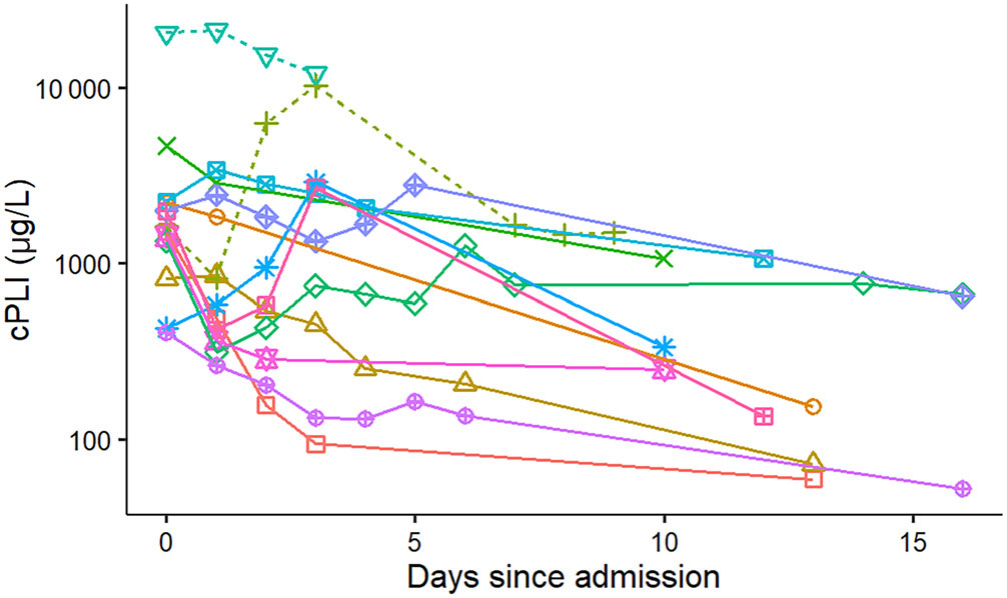
Change in serum canine pancreatic lipase immunoreactivity (cPLI) concentrations over time in 13 hospitalized dogs with pancreatitis from a single referral hospital. Solid lines symbolize surviving dogs and dashed lines indicate dogs that died or were euthanized

**FIGURE 4:**
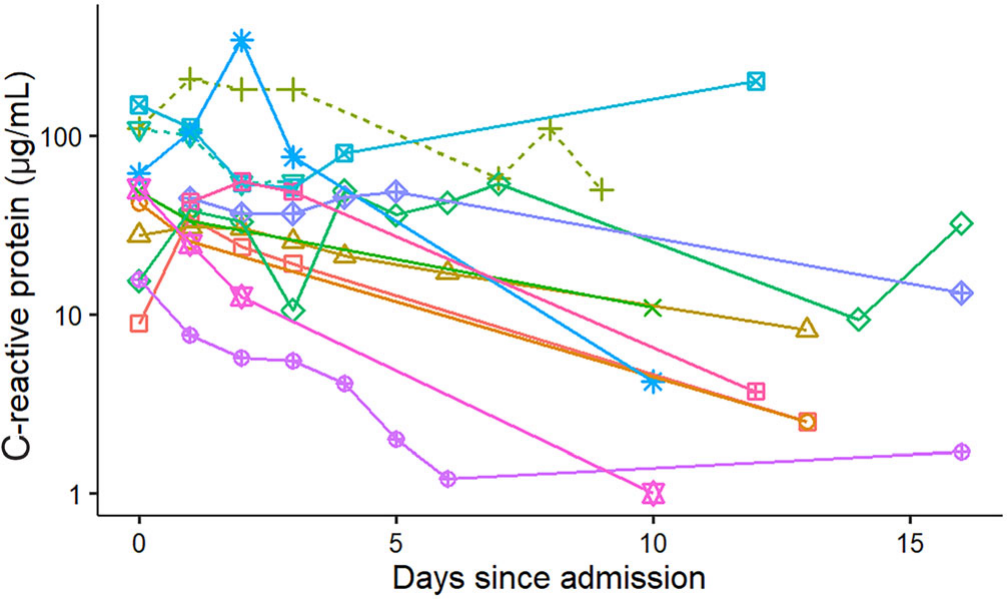
Change in serum C‐reactive protein concentrations over time in 13 hospitalized dogs with pancreatitis from a single referral hospital. Solid lines symbolize surviving dogs and dashed lines indicate dogs that died or were euthanized

The majority of surviving dogs showed a decrease of both serum cPLI and CRP concentrations during hospitalization and at re‐evaluation. Of the 2 nonsurviving dogs, the dog that was euthanized showed improvement in both serum cPLI and CRP concentrations, whereas the dog that died had serum cPLI and CRP concentrations that fluctuated throughout hospitalization. However, both nonsurvivor dogs had quantitatively higher serum cPLI (Figure 3) and CRP (Figure 4) concentrations than the majority of surviving dogs.

**FIGURE 5:**
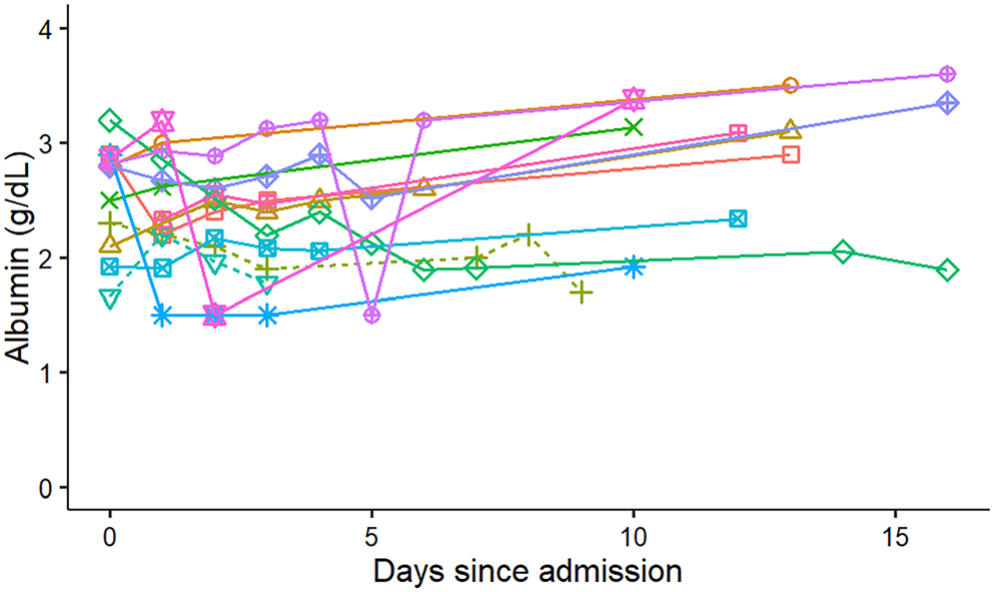
Change in serum albumin concentrations in 13 hospitalized dogs with pancreatitis from a single referral hospital. Solid lines symbolize surviving dogs and dashed lines indicate dogs that died or were euthanized

The majority of surviving dogs had serum albumin concentrations that increased from the time of the initial diagnosis to re‐evaluation. In contrast, both nonsurviving dogs had serum albumin concentrations that decreased before death. Nonsurvivors had quantitatively lower serum albumin concentrations than the majority of surviving dogs (Figure 5).

**FIGURE 6:**
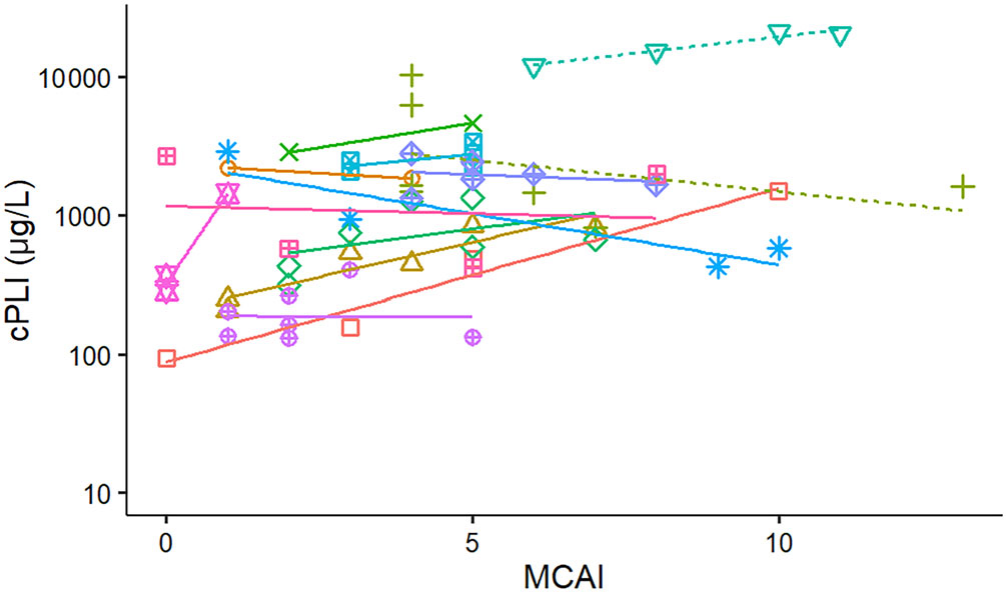
Correlation between canine pancreatic lipase immunoreactivity (c‐PLI) and modified canine activity index (MCAI) from diagnosis to discharge or death in 13 dogs with pancreatitis admitted to a single referral hospital. Solid lines symbolize surviving dogs and dashed lines indicate dogs that died or were euthanized

In the majority of surviving dogs, serum cPLI concentrations increased with increasing MCAI clinical scores (Figure 6) and these variables were significantly correlated after adjusting for the repeated measures sampling design in the survivor group, and when combining both the survivors and nonsurvivors into a single group (Table 3). Of the 2 nonsurvivors, the dog that was euthanized had an improved MCAI severity score as the serum cPLI concentration decreased. However, the dog that died initially had improvement in the MCAI severity score and a lower serum cPLI concentration, but then the MCAI severity score and serum cPLI concentration plateaued the last few days before death.

**TABLE 3:** The correlation between MCAI and CAPCSI with canine pancreatic lipase immunoreactivity (cPLI), c‐reactive protein (CRP), and albumin concentration from admission until discharge or death in 11 surviving and 2 nonsurviving dogs hospitalized for pancreatitis

Dog population	Clinical score	Biomarker	n^a^	Spearman's rho (*P* value)	Model correlation^b^ (*P* value)
All dogs	MCAI	cPLI	62	0.417 (.001)	0.417 (.01)
		CRP	60	0.433 (.001)	0.414 (.02)
		Albumin	61	−0.181 (.16)	−0.008 (.95)
	CAPCSI	cPLI	62	0.266 (.04)	0.146 (.35)
		CRP	60	0.214 (.1)	0.262 (.15)
		Albumin	61	−0.222 (.09)	−0.309 (.01)
Surviving	MCAI	cPLI	51	0.331 (.02)	0.330 (.02)
		CRP	49	0.295 (.04)	0.310 (.1)
		Albumin	50	−0.077 (.6)	0.001 (1)
	CAPCSI	cPLI	51	0.177 (.21)	0.117 (.49)
		CRP	49	0.266 (.06)	0.383 (.07)
		Albumin	50	−0.155 (.28)	−0.299 (.03)
Died/euthanized	MCAI	cPLI	11	0.312 (.35)	−0.547 (.14)
		CRP	11	0.089 (.8)	0.317 (.28)
		Albumin	11	0.306 (.36)	0.573 (.03)
	CAPCSI	cPLI	11	0.689 (.02)	−0.164 (.55)
		CRP	11	−0.592 (.06)	−0.388 (.04)
		Albumin	11	−0.454 (.16)	−0.120 (.56)

Abbreviations: CAPCSI, canine acute pancreatitis clinical severity index; cPLI, canine pancreatic lipase immunoreactivity; CRP, C‐reactive protein; MCAI, modified canine activity index.

^a^ Total number of data points from diagnosis until discharge or death with multiple data points per dog.

^b^ Estimated through the use of a mixed‐effects linear regression model accounting for individual dog effects.

**FIGURE 7:**
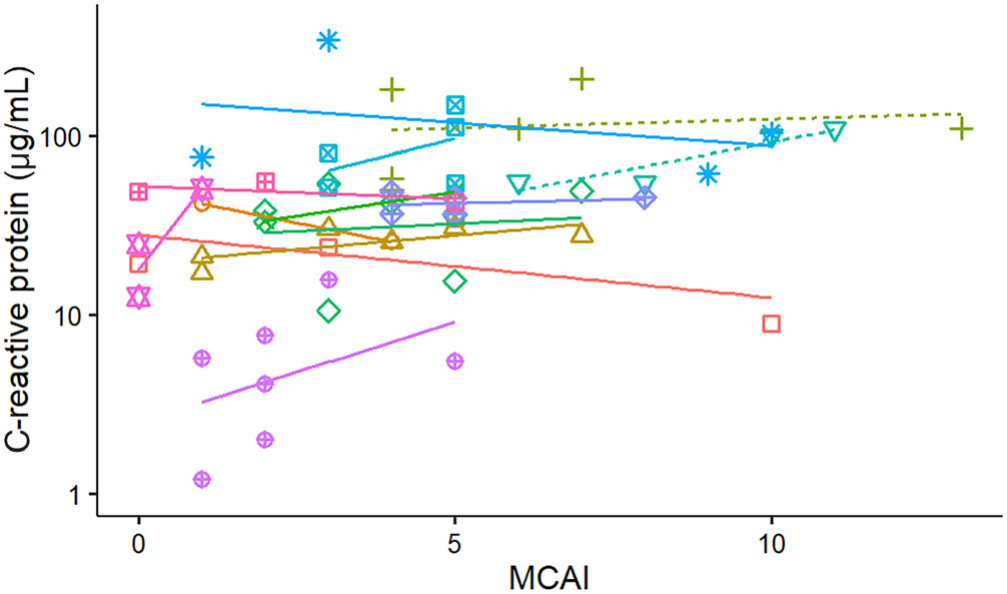
Correlation between serum C‐reactive protein concentration and modified canine activity index (MCAI) from diagnosis to discharge or death in 13 dogs with pancreatitis admitted to a single referral hospital. Solid lines symbolize surviving dogs and dashed lines indicate dogs that died or were euthanized

The CRP concentrations also increased with MCAI severity score in both survivors and nonsurvivors (Figure 7), but the positive correlation was not significant after adjustment for the repeated sampling design when evaluating the survivor group individually (*P* = .1). However, when evaluating all dogs together, a significant correlation was observed between serum CRP concentrations and the MCAI severity score (*P* = .01).

No relationship was apparent between serum albumin concentration and the MCAI in survivors (data not presented) but a significant positive correlation was observed between these variables in nonsurvivors (*P* = .03). In the patient that was euthanized, the serum albumin concentration improved initially but then continued to decrease despite a decreasing MCAI. In the patient that died, the serum albumin concentration fluctuated similar to the MCAI, until the day it died when the serum albumin concentration decreased substantially with a mild decrease in the MCAI.

**FIGURE 8:**
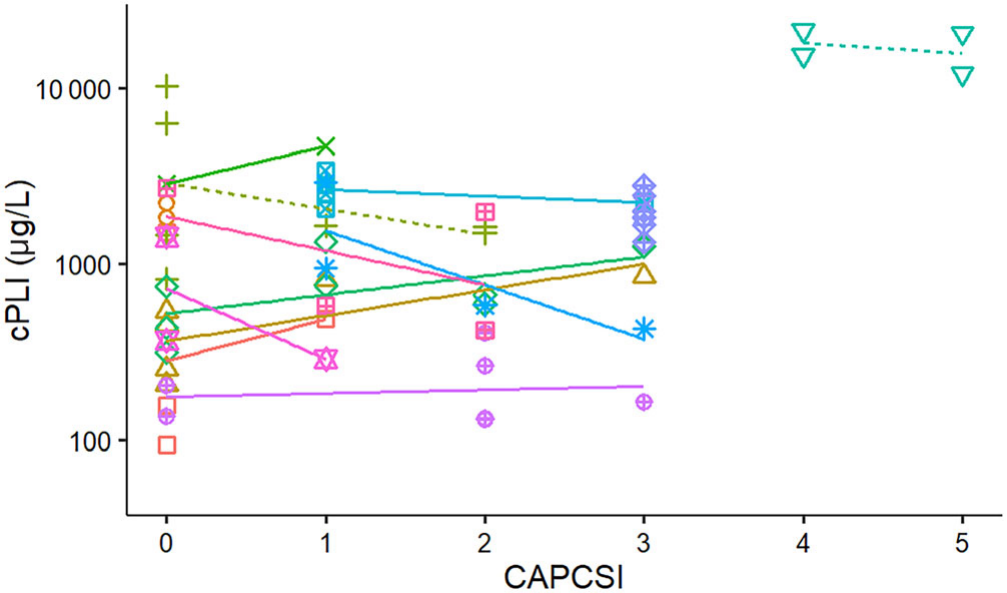
Correlation between canine pancreatic lipase immunoreactivity (c‐PLI) and CAPCSI from diagnosis to discharge or death in 13 dogs admitted to a single referral hospital. Solid lines symbolize surviving dogs and dashed lines indicate dogs that died or were euthanized. CAPCSI, canine acute pancreatitis clinical severity index

**FIGURE 9:**
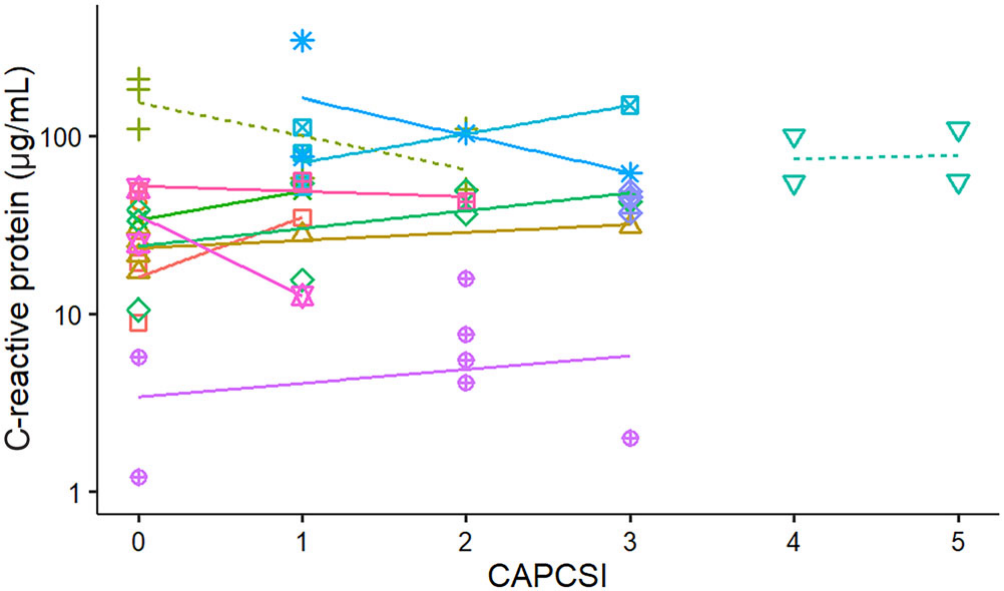
Correlation between serum C‐reactive protein concentration and CAPCSI from diagnosis to discharge or death in 13 dogs admitted to a single referral hospital. Solid lines symbolize surviving dogs and dashed lines indicate dogs that died or were euthanized. CAPCSI, canine acute pancreatitis clinical severity index

No significant relationship was found between serum cPLI concentrations and CAPCSI (Figure 8), nor between serum CRP concentrations and CAPCSI (Figure 9) in both survivor and nonsurvivor groups, or when all dogs were evaluated together.

## 4 DISCUSSION

We hypothesized that serum CRP and cPLI concentrations would decrease during hospitalization in conjunction with clinical improvement, as dogs recovered from acute pancreatitis as assessed using the MCAI and the CAPCSI. The MCAI clinical scores differed between survivors and nonsurvivors, suggesting that this scale might be more useful for determining prognosis than the CAPCSI in this group of patients. One possible reason for this difference was the fact that CAPCSI scores overall were very low, whereas the range of MCAI scores was larger. Also, a significant correlation was observed between MCAI scores and serum cPLI concentrations when evaluating both survivor and nonsurvivor groups combined, as well as when evaluating the survivor group alone. As the clinical severity score as assessed by the MCAI improved, serum cPLI concentrations decreased. This finding suggests that in hospitalized dogs with acute pancreatitis, decreasing serum cPLI concentration is associated with better clinical outcome.

A significant correlation also was found between the MCAI and serum CRP concentration when both the survivor and nonsurvivor groups were combined. Although serum CRP concentration appears to be a useful biomarker to identify clinical changes in hospitalized dogs with acute pancreatitis, the decrease in serum cPLI was more consistent on a daily basis as compared to serum CRP concentration. Additionally, unlike serum cPLI, serum CRP concentration was not significantly correlated with MCAI when evaluating the survivor group alone. Serum CRP concentration is affected by a wide variety of conditions of different organs,^6^, ^7^, ^8^, ^9^, ^10^, ^11^, ^14^, ^15^, ^16^, ^17^, ^19^, ^21^ whereas serum cPLI is specific for acinar cell damage and pancreatitis. Many dogs in our study had concurrent diseases, so it is difficult to determine whether serum CRP concentration was affected by acute pancreatitis or the concurrent conditions. Serum CRP concentration might be more useful in dogs with pancreatitis but without concurrent conditions or complications. However, it is not uncommon for dogs to have at least 1 other concurrent condition or systemic complication in addition to moderate or severe pancreatitis, making it difficult to determine a correlation between CRP and improving or worsening pancreatitis. Another possible contributing factor as to why serum CRP concentration was not significantly correlated with the MCAI in surviving dogs is duration of hospitalization. A previous study found that serum CRP concentration was not significantly different between the surviving and nonsurviving groups in dogs with acute pancreatitis until the third and fourth day of hospitalization.^31^ Additionally, although serum CRP concentration decreases rapidly, it can take from 3 to 14 days to normalize even if no other trigger for CRP release is present.^32^ Six of the 11 surviving dogs in our study were discharged after 2 to 4 days of hospitalization, and the remaining 5 were discharged after 6 to 8 days of hospitalization. Because of the small sample size and short duration of hospitalization in 54% of the survivors, it could be more difficult to detect a significant change in serum CRP concentration.

The CAPCSI severity scores were not different between dogs that recovered from acute pancreatitis and those that died. This finding could be related to small sample size but CAPCSI only assesses 4 clinical variables (ie, cardiac changes, respiratory changes, intestinal integrity, and blood pressure) and all would only be expected to be abnormal in dogs with more severe forms of pancreatitis. In contrast, the MCAI assesses 7 clinical variables (ie, activity, appetite, vomiting, cranial abdominal pain, dehydration, feces consistency, and presence or absence of blood in the feces) that assess more common clinical signs in dogs with pancreatitis. The difference in scale usefulness was demonstrated by our finding that the MCAI score clearly changed over time in the dogs with pancreatitis during hospitalization, whereas the pattern of the CAPCSI scores was inconsistent within the same dogs. Even the nonsurvivors had few changes in the CAPCSI scores and the CAPCSI scores did not differ significantly between survivors and nonsurvivors. In this group of dogs, the CAPCSI score did not appear to be useful for evaluating severity of clinical disease, but it is possible that a study of a larger number of dogs with more severe disease might identify differences. Because only 2 dogs did not survive, we were unable to evaluate whether a significant correlation existed between the MCAI and serum CRP concentration or cPLI in the nonsurviving dogs. However, both the MCAI and serum cPLI decreased in the dog that was euthanized. If this dog had continued treatment rather than being euthanized, the clinical outcome might have been different. However, in the dog that died, the MCAI increased as serum cPLI decreased. One possible explanation for this finding is pancreatic necrosis and exhaustion of acinar cells, thereby decreasing the amount of lipase leaking from the acinar cells into the vascular space, but further morphological and ultrastructural studies in dogs with similar findings are needed to evaluate this hypothesis further.

We were unable to identify a significant correlation between the MCAI and serum albumin concentrations when evaluating any of the groups. However, the majority of the surviving dogs had improvement in their serum albumin concentrations as the MCAI decreased. This is not unexpected because albumin is a known negative acute phase protein that decreases with acute inflammation, including acute pancreatitis.^5^ A study of a larger number of survivor and nonsurvivor dogs would be necessary to further evaluate the association between MCAI and serum albumin concentration in dogs with acute pancreatitis.

The correlation between MCAI and serum cPLI or CRP concentrations was not perfect (Figures 6 and 7). There are several possible reasons for this observation. First, several of the dogs had low MCAI scores for several time points and thus changes of serum concentrations of both potential biomarkers could be interpreted as a lack of correlation. However, it is possible that these biomarkers simply are more sensitive than assigning an MCAI score and further studies using a larger number of dogs would be required to investigate this possibility further. Overall congruency of lines for cases between Figures 1 and 3 for cPLI and between Figures 1 and 4 for CRP suggests that these variables agree better than the correlation depicted in Figures 6 and 7 would suggest. Also, clinical findings and serum concentrations of biomarkers might not follow the same time line (ie, changes in biomarkers may occur after a delay). Again, this possibility can be better assessed when comparing Figures 1 and 3 or Figures 1 and 4 because clinical signs as assessed by the MCAI improve faster than biomarker concentrations. Again, this finding deserves further investigation in a larger number of dogs.

One may consider why the use of objective biomarkers in dogs with pancreatitis is necessary when the MCAI appears to provide information about disease progression. Certainly, our study did not provide sufficient data to suggest that serum cPLI or CRP concentration should replace clinical assessment. However, clinical studies of dogs with pancreatitis, including studies of new therapeutic targets, require both clinical scoring systems as well as objective biomarkers for the appropriate assessment of outcomes.

Our study had several limitations. Pancreatic biopsy was not performed in any of the dogs, and therefore pancreatitis could not be confirmed histopathologically. However, collection of a pancreatic biopsy sample is invasive and could not be justified clinically in any of the enrolled dogs. Also, our study used strict diagnostic criteria for classification of pancreatitis, and we therefore are confident of our diagnosis in all study dogs. A second limitation was the limited number of cases, and especially of cases in the nonsurvivor group. Furthermore, 1 of the dogs in the nonsurvivor group was euthanized and thus it is unknown if that dog would have eventually recovered. The 1 patient that died had variable serum concentrations of both cPLI and CRP, whereas the patient that was euthanized showed improving serum cPLI and CRP concentrations as well as an improving MCAI. A larger study with more patients would have increased our statistical power to identify significant relationships. Another limitation is that neither the MCAI nor the CAPCSI scores have been validated for the clinical progression of pancreatitis in dogs. To our knowledge, ours is the first study in which the MCAI and CAPCSI scores have been used for the purpose of monitoring disease progression. More studies are needed to determine the validity and use of these scoring systems to assess the clinical status of dogs with pancreatitis. Last, although we attempted to have the same clinician examine each patient over time, doing so was not always possible. Differences in opinion relating to clinical observation could have affected the MCAI and CAPCSI scores in some patients.

Our results provide additional information for the management of hospitalized dogs with acute pancreatitis and suggest that serum cPLI and CRP concentration might serve as objective markers of disease progression. Consistently increasing serum cPLI, CRP concentration, or both despite aggressive management may help ease owner concern about discontinuing treatment efforts. Additionally, it might prompt clinicians to adjust their current management.

In conclusion, the MCAI improved over time in most hospitalized dogs with acute pancreatitis that survived to discharge, whereas the CAPCSI within the same group of dogs did not demonstrate changes over time. Also, serum cPLI concentrations correlated with clinical severity both in surviving dogs and in all dogs with pancreatitis. Serum CRP concentrations correlated with clinical severity in all dogs with pancreatitis, but not in surviving dogs with pancreatitis. These results suggest that serum cPLI and potentially CRP concentrations could be used as objective biomarkers for disease progression and monitoring management success in dogs with acute pancreatitis. Additional studies involving larger numbers of dogs are warranted to evaluate the impact of these findings on the management of dogs with acute pancreatitis.

## CONFLICT OF INTEREST DECLARATION

Dr Joerg M. Steiner serves as the Director of the Gastrointestinal Laboratory, Texas A&M University, which performs cPLI and C‐reactive protein testing on a fee‐for‐service basis. Dr Joerg M. Steiner also serves as a paid consultant for Idexx Laboratories, the manufacturer of Spec cPL. No other authors have a conflict of interest to declare.

## OFF‐LABEL ANTIMICROBIAL DECLARATION

Authors declare no off‐label use of antimicrobials.

## INSTITUTIONAL ANIMAL CARE AND USE COMMITTEE (IACUC) OR OTHER APPROVAL DECLARATION

Authors declare no IACUC or other approval was needed.

## HUMAN ETHICS APPROVAL DECLARATION

Authors declare human ethics approval was not needed for this study.
